# Traumatic index extensor tendon attenuation mimicking closed tendon rupture: two case reports

**DOI:** 10.1186/s12891-020-03692-6

**Published:** 2020-10-10

**Authors:** Yusuke Miyashima, Takuya Uemura, Takuya Yokoi, Shunpei Hama, Mitsuhiro Okada, Sadahiko Konishi, Hiroaki Nakamura

**Affiliations:** 1 Department of Orthopedic Surgery, Osaka General Hospital of West Japan Railway Company, 1-2-22 Matsuzakicho, Abeno-ku, Osaka, 545-0053 Japan; 2 Department of Orthopedic Surgery, Osaka City University Graduate School of Medicine, 1-4-3, Asahimachi, Abeno-ku, Osaka, 545-8585 Japan

**Keywords:** Extensor tendon rupture, Index finger, Attenuation, Wide awake surgery, Pseudorupture

## Abstract

**Background:**

While some traumatic closed index extensor tendon ruptures at the musclotendinous junction have been previously reported, closed index extensor tendon pseudorupture due to intertendinous attenuation is exceedingly rare with only one case report of a gymnastics-related sports injury in the English literature. Herein, we report two non-sports injury related cases of traumatic index extensor tendon attenuation mimicking closed tendon rupture, including the pathological findings and intraoperative video of the attenuated extensor indicis proprius tendon.

**Case presentation:**

A 28-year-old man and a 30-year-old man caught their hands in a high-speed drill and lathe, respectively, which caused a sudden forced flexion of their wrists. They could not actively extend the metacarpophalangeal joints of their index fingers. Intraoperatively, although the extensor indicis proprius and index extensor digitorum communes tendons were in continuity without ruptures, both tendons were attenuated and stretched. The attenuated index extensor tendons were reconstructed either with shortening by plication or step-cut when the tendon damage was less severe or, in severely attenuated tendons, with tendon grafting (ipsilateral palmaris longus) or tendon transfer. Six months after the operation, the active extension of the index metacarpophalangeal joints had recovered well.

**Conclusions:**

Two cases of traumatic index extensor tendon attenuation were treated successfully by shortening the attenuated tendon in combination with tendon graft or transfer. We recommend WALANT (wide-awake local anesthesia and no tourniquet) in the reconstruction surgery of index extensor tendon attenuation to determine the appropriate amount of tendon shortening or optimal tension for tendon grafting or transfer. Intraoperative voluntary finger movement is essential, as it is otherwise difficult to judge the stretch length of intratendinous elongation and extent of traumatic intramuscular damage affecting tendon excursion.

## Background

Subcutaneous extensor tendon rupture is usually caused by tendon attrition or friction in association with rheumatoid arthritis or osteoarthritis of the distal radioulnar joint, and distal radius fracture [1, 2]. However, traumatic closed extensor tendon rupture of the index finger alone is uncommon and in previous reports has typically occurred at the musclotendinous junction [1–5]. Particularly, closed index extensor tendon pseudorupture due to intertendinous attenuation is very rare and has only been described once in a case report of gymnastics induced injury in the English literature [6]. Herein, we report two non-sports related injuries of traumatic index extensor tendon attenuation mimicking closed tendon rupture, in which the pathological finding and intraoperative video of the attenuated tendons were documented. The patients were then treated successfully by shortening the attenuated extensor tendon in combination with tendon graft or transfer.

## Case presentation

### Case 1

**Fig. 1:**
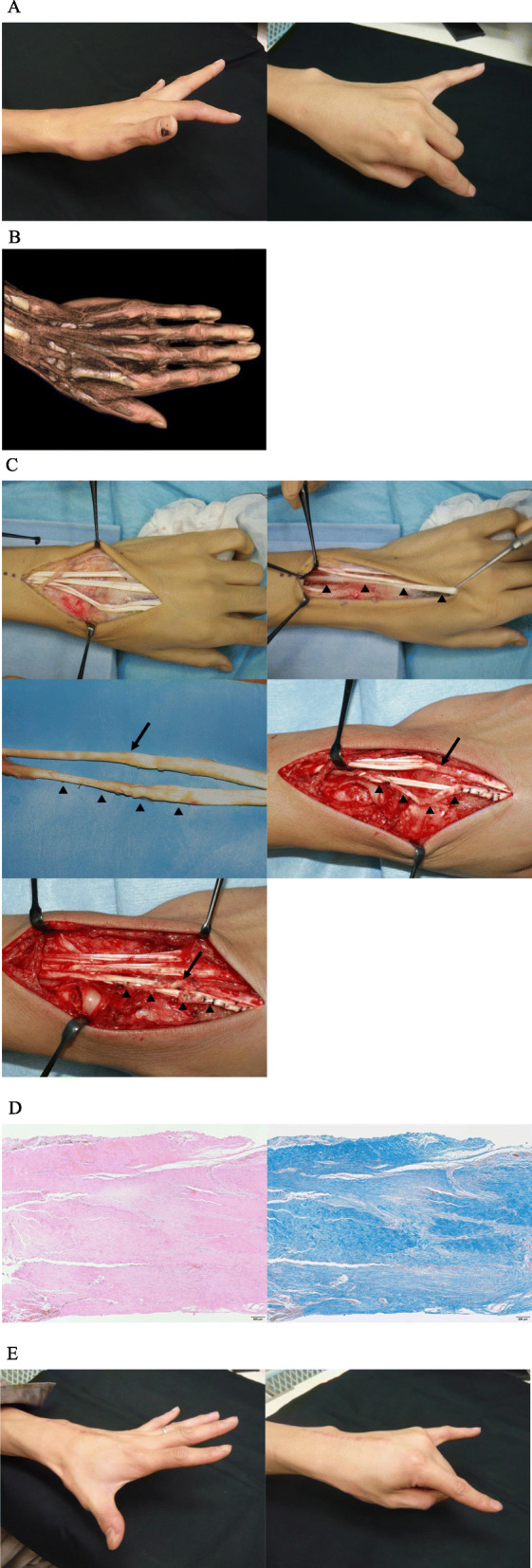
**a**. Preoperative extension loss of the MP joint of the left index finger. **b**. Three-dimensional CT imaging showing the slackness of the index EDC and EIP tendons. **c**. Intraoperative findings of the attenuated tendons of both the EIP (black triangle) and index EDC (black arrow) without tendon ruptures at the muscloctaneous junction. Repairing the attenuated EIP tendon with palmaris longus tendon grafting and plication of the attenuated index EDC tendon. **d**. Hematoxylin and eosin staining and Masson trichrome staining of the attenuated EIP tendon showing continuity of the tendon with some intratendinous microruptures. **e**. Postoperative extension of the MP joint of left index finger

A 28-year-old man caught his work glove in a high-speed drill which caused a sudden forced flexion of his left wrist. He experienced severe dorsal wrist pain at the time of injury, and he was unable to extend his index finger. At his first visit to our clinic 6 weeks after the injury, he could not actively extend the metacarpophalangeal (MP) joint of his index finger while he could fully extend the others (Fig. 1). There was no laboratory evidence of rheumatoid arthritis or inflammatory arthropathy. Radiographs showed neither fractures nor osteophytes in the forearm and hand. On three-dimensional computed tomography (CT, Volume Analyzer SYNAPSE VINCENT, Fujifilm, JAPAN) images, the index extensor digitorum communes (EDC) tendon seemed to be in continuity, but slack, and the extensor indicis proprius (EIP) tendon was poorly defined. Magnetic resonance imaging (MRI) and ultrasonography showed extensor tenosynovitis in the fourth extensor compartment of the dorsal wrist. Muscular denervation of EDC and EIP was not detected on electromyography. As traumatic subcutaneous EIP tendon rupture was suspected, surgery under general anesthesia was performed. At exploration via a 10-cm longitudinal dorsal incision, the extensor tendons involved severe tenosynovitis and complete tenolysis was performed. Although the EIP and index EDC tendons were in continuity without ruptures, both tendons were attenuated and stretched at zone VI. Neither tendon showed evidence of an avulsion rupture at the musculotendinous junction. The attenuated lesion of the EIP tendon was resected and reconstructed with a tendon graft of the ipsilateral palmaris longus (PL) because the attenuated EIP tendon had suffered severe damage and was too fragile to be reused. The attenuated index EDC tendon was reconstructed with shortening of 1 cm by plication at the distal to the extensor retinaculum as the tendon damage was less severe. Pathological findings of the resected EIP tendon showed collagen fibers arrayed irregularly involving synovial proliferation with lymphocytes repairing intertendinous microruptures without neutrophilic infiltration. After the operation, early active range of motion exercise was initiated with dynamic outrigger splinting. Six months after the operation, the active extension of the index MP joint had recovered. The patient-reported outcomes measured by the Quick Disability of the Arm, Shoulder, and Hand (Quick DASH) and the Hand 20 were 20.5 and 12.5, respectively.

### Case 2

**Fig. 2:**
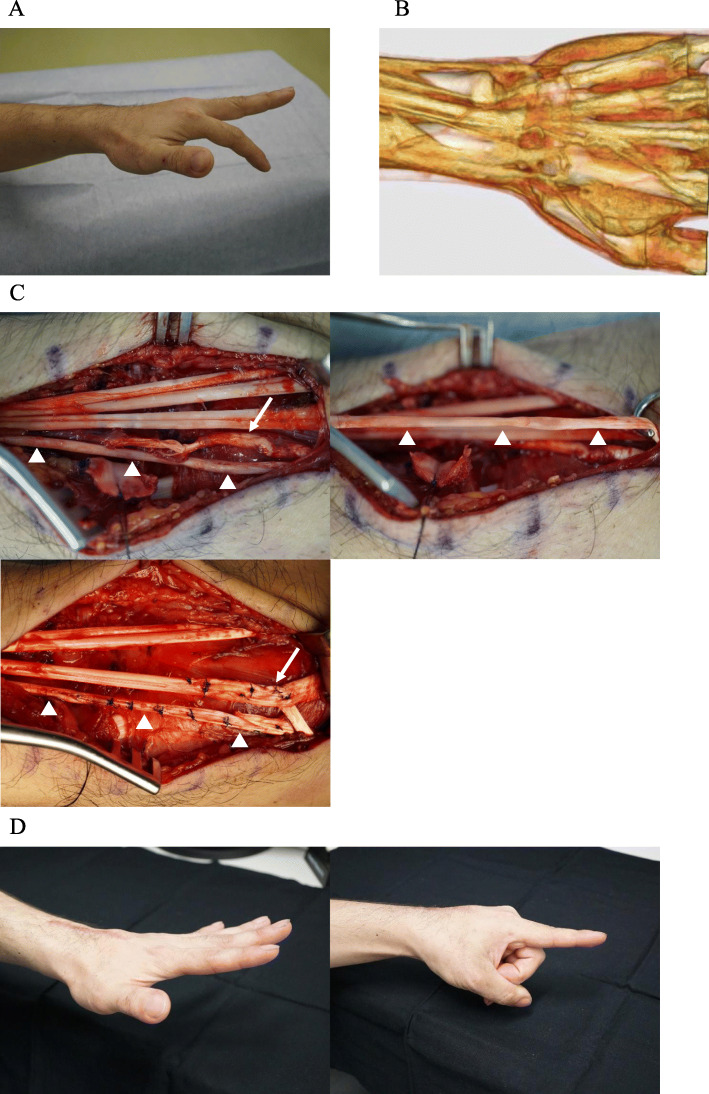
**a**. Preoperative extension loss of the MP joint of the left index finger. **b**. Three-dimensional CT imaging showing the slackness of the index EDC and EIP tendons. **c**. Intraoperative finding of the attenuated EIP tendon (white triangle) and partial tear of the index EDC tendon (white arrow). Shortening of the attenuated EIP tendon and tendon transfer of the index EDC to the middle EDC tendon. **d**. Postoperative full extension of the MP joint of the left index finger

While working, a 30-year-old man caught his left hand in the lathe and this caused a sudden forced flexion of his left wrist with pain. After that, he could not extend his index finger. At his first visit to our clinic 2 weeks after the injury, he could not extend the MP joint of his left index finger (Fig. 2). On radiographs and CT images, a small avulsion fracture of the dorsal aspect of the trapezoid was found. On three-dimensional CT images, both the index EDC and EIP tendons were serpentine and slack, yet with preserved continuity. The MRI and ultrasonography showed substantial extensor tenosynovitis on the dorsal wrist. As traumatic subcutaneous index extensor tendon ruptures were suspected, WALANT (wide-awake local anesthesia and no tourniquet) surgery was performed. At exploration, the index EDC tendon was partially ruptured with elongation and the EIP tendon was attenuated without rupture at zone VI. Because tendon damage of the index EDC was severe, the index EDC tendon was transferred to the middle EDC tendon. The attenuated EIP tendon was reconstructed with utilization of step-cut shortening by two centimeters at the distal to the extensor retinaculum. During these tendinous reconstruction surgeries, we determined an appropriate tension level with the voluntary movement of the patient’s index finger (Supplemental file, Video). After the operation, early active range of motion exercise was initiated with buddy taping of the index and middle fingers. Six months after the operation, the active extension of the index MP joint had recovered fully. The scores of the Quick DASH and Hand 20 were 2.3 and 2, respectively.

## Discussion and conclusion

Acute closed extensor tendon pseudorupture due to intratendinous attenuation is very rare [6, 7]. Adler et al. reported a case of an 18-year-old man with traumatic intratendinous attenuation of the extensor pollicis longus (EPL) tendon after a fall in which a 5-cm segment of the EPL tendon just distal to the musclotendinous junction was attenuated and stretched intraoperatively [7]. Sathyendra and Payatakes reported a case of a 24-year-old gymnast with intratendinous attenuation of the EIP and EDC to the index, long, and ring fingers tendons after a grip lock injury using the horizontal bar in gymnastics [6]. Grip lock injuries typically cause excessive tensile forces on dorsal aspects of the wrist in male gymnasts. This occurs when a poorly fitted or stretched-out leather grip completely encircles the high bar and locks onto itself in the giant swing [6]. In the present two cases of non-sports related injury, similar high tensile forces to the grip lock might be applied on the dorsal aspect of the wrist after the index finger was caught in the high-speed drill or lathe, resulting in tendon attenuation of only that finger.

In vivo research of tendon biomechanics using a human anterior cruciate ligament, it was revealed that the mechanical behavior of the tendon depended on the rate of mechanical strain because tendons have fiber patterns and viscoelastic characteristics [8–10]. According to the tendon stress-strain curve, a tendon is stretched when tensile forces work on the tendon and the tendon is strained up to 4%. Then, microscopic tearing of the tendon fibers occurs when the strain is greater than 4%. Macroscopic tearing of the tendon occurs when the strain is increased beyond 8–10% and eventually, further strain caused tendon rupture. In the present cases, the EIP tendon attenuation might occur when 4 to 8% strain force is created by catching the index finger in the high-speed drill or lathe. These speculations were supported by the pathological findings of the attenuated EIP tendon that had microscopic tears and evidence of an intrinsic healing process taking place with proliferation of lymphocytes and tenocytes. There has been no histological evaluation of the attenuated extensor tendon in previous reports [6, 7]. Moreover, the three-dimensional reconstructed CT images of the extensor tendons are also useful for the assessment of the extensor tendon attenuation [4]. Ultrasonographic images are also useful for the assessment of real-time tendon excursion and rupture. On three-dimensional CT and ultrasonographic images, macro irregularity of the extensor tendon such as rupture, slackness, and redundancy is able to be detected but it is difficult to differentiate between attenuation and micro-rupture.

It is still controversial which is the best surgical method: tendon shortening or tendon grafting and transfer for the reconstruction of the attenuated tendon. In the previous report of the attenuated EPL tendon, the region of attenuation was transected and imbricated [7]. In another report of the attenuated EIP and EDC tendons in a gymnast, an imbrication of the affected tendons with a Z-configuration was performed [6]. In the present case 1, the attenuated EIP and index EDC tendons were reconstructed with grafting of a PL tendon and a shortening by plication, respectively. In the second case, the attenuated EIP and index EDC tendons were reconstructed with step-cut shortening similar to the previous gymnast case and tendon transfer to the middle EDC. Our surgical strategy for the reconstruction of the attenuated tendon was to perform tendon shortening by plication or imbrication. This method should be chosen when the damage and fragility of the attenuated tendon is slight to mild, and tendon graft or transfer should be chosen alternatively if the damage and fragility of the attenuated tendon is too severe to reuse it. Herein, it is very difficult to determine the appropriate amount of tendon shortening and tension for tendon reconstruction because we cannot fully detect the stretched length of the elongated tendon nor gauge the extent of traumatic intramuscular damage affecting tendon excursion. Sathyendra and Payatakes also advised that the surgeon must use judgment based on intraoperative findings under a general anesthesia to achieve the optimal balance between extensor lag and extrinsic extensor tightness [6]. Therefore, we recommend that WALANT is most suitable for the reconstruction of extensor tendon attenuation, as the present case 2 (the intraoperative video shown above) resulted in a more normalized extension of the index finger alone and better improvement in the Quick DASH and Hand 20 scores than the present case 1 under general anesthesia. It is important to assess the extent of the intratendinous elongation and tendon excursion like in vivo and to decide the best surgical method including the appropriate amount of tendon shortening or optimal tension for tendon grafting or transfer under intraoperative voluntary finger movement in the surgery of index extensor tendon attenuation.

## Supplementary information

**Additional file 1.**

## Abbreviations

MP: Metacarpophalangeal
CT: Computed tomography
EDC: Extensor digitorum communes
EIP: Extensor indicis proprius
MRI: Magnetic resonance imaging
DASH: Disability of the Arm Shoulder and Hand
EPL: Extensor pollicis longus
